# A Scoping Review of Foot‐and‐Mouth Disease in Wildlife: Global Evidence to Inform Preparedness in Nonendemic Regions

**DOI:** 10.1155/tbed/9482115

**Published:** 2026-11-01

**Authors:** Emma G. Gardner, Erin N. Casper, Murray Gillies, Nicole Wanamaker, Jamie L. Rothenburger

**Affiliations:** ^1^ Animal Health Canada Elora Ontario Canada; ^2^ Faculty of Veterinary Medicine and Alberta Region of the Canadian Wildlife Health Cooperative University of Calgary Calgary Alberta Canada ucalgary.ca; ^3^ New Brunswick Department of Agriculture, Aquaculture and Fisheries Sussex New Brunswick Canada

**Keywords:** disease control strategies, spillover risk, transboundary animal disease, wildlife-livestock interface, wildlife surveillance

## Abstract

Foot‐and‐mouth disease (FMD) is a highly contagious virus with severe economic and animal welfare consequences if introduced into nonendemic regions. Given its broad host range, including domestic and wild animals, wildlife must be considered in FMD preparedness and response planning. We conducted a scoping review according to PRISMA‐ScR guidelines and narrative synthesis of 205 publications (year 2000 onward) to examine wildlife susceptibility to FMD and associated control strategies. In the Canadian context, over a dozen native wild ungulate species, as well as feral pigs, are known or likely to be susceptible to FMD. Experimental studies confirm that species such as deer, bison, and pronghorn can develop clinical disease and transmit FMD, yet their potential role as long‐term maintenance hosts remains unclear. This knowledge gap highlights the critical need for targeted research to inform surveillance and control strategies in disease‐free regions. Globally, wildlife‐livestock interfaces are recognized as critical points for potential FMD transmission. Surveillance typically relies on opportunistic sampling of hunted or found‐dead animals. Modeling studies suggest that outbreaks in wildlife are generally self‐limiting without livestock involvement, constrained by ecological factors such as seasonality, population density, and short infectious periods. However, environmental persistence and shared resource use (e.g., water and feed) may facilitate transmission. Control measures include barrier fencing and livestock ring vaccination. Wildlife culling is widely considered infeasible, environmentally damaging, and socially contentious, with use recommended only under narrow, evidence‐based conditions. Enhanced biosecurity, such as reducing wildlife attractants on farms, and movement restrictions are consistently emphasized. Preparedness in nonendemic regions should prioritize risk‐based wildlife surveillance, cross‐sectoral coordination, response protocols, and strategies to mitigate impacts on vulnerable species. An integrated approach encompassing ecological, wildlife, and agricultural perspectives is essential for coordinated, science‐based responses to future FMD incursions involving wildlife.

## 1. Introduction

Foreign animal diseases (FADs) are highly contagious diseases that are not normally present in a country’s animal population. As such, FADs can have significant and disruptive economic, public health, and social consequences. To prepare for possible introduction of FADs into Canada, the Canadian Food Inspection Agency (CFIA) has designated specific diseases as priorities to strengthen contingency planning [1]. Among these diseases is foot‐and‐mouth disease (FMD), caused by FMD virus (FMDV), a highly contagious pathogen in the genus *Aphthovirus* of the family *Picornaviridae* [2]. The virus is assumed to affect all members of the order Artiodactyla, most commonly infecting cattle, swine, small ruminants, and other cloven‐hooved animals such as deer and bison [3]. The typical disease presentation involves vesicles in and around the mouth, causing excessive salivation, and on the hooves within the interdigital space and around the coronary band, leading to lameness [4, 5]. Lesions can vary significantly between species but are commonly accompanied by nonspecific clinical signs including fever, depression, lethargy, and reduced production in livestock [5]. Because FMD is highly transmissible with significant production loss, it is one of the most important livestock diseases globally [6] and is a federally reportable disease in Canada. While it has not been detected in the country since 1952 [7], if the disease were to occur again, there would be devastating impacts on the livestock industry [8, 9], international trade agreements [6], and both human and animal welfare [10, 11].

Historically, research and control strategies focused solely on domestic livestock, as wild species were not believed to play a significant role in the transmission of the disease outside of southern Africa [12]. But land‐use changes have increased wildlife‐livestock interactions, bringing greater attention to the potential role of wildlife in FMD epidemiology [13]. The World Organization for Animal Health (WOAH) emphasizes that effective management of transboundary animal diseases requires explicit consideration of the wildlife‐livestock interface, including surveillance, risk assessment, and coordinated response across sectors [14]. Understanding FMDV epidemiology, including the roles and susceptibility of wildlife species, is essential to developing holistic management strategies that ensure the safety and well‐being of both domestic livestock and wildlife populations.

There remain significant challenges to enhancing our knowledge of the relationship between wildlife and FMD. With over 100 documented species of wildlife with varying degrees of susceptibility, only a few species of free‐ranging populations have been extensively studied [15]. Some species may act as a reservoir, while others experience high levels of mortality [16]. The roles wildlife populations play in the ecology of FMD are diverse, and while Canada is home to at least 14 free‐ranging ungulate species and numerous other potentially susceptible species [17], their potential role in an outbreak remains unknown. Moreover, there is no recent literature that explores the potential impact of FMD in wild animals in nonendemic areas or summarizes prevention and control measures that target wild animal populations, including the efficacy of these measures. The aim of this review is to synthesize and describe the existing knowledge base and expert recommendations regarding the role of wildlife in FMD epidemiology, ecology, and control through both peer‐reviewed and gray literature, with the specific lens of applicability to jurisdictions where FMD is considered a FAD. The specific objectives of this review are (i) to identify and summarize the empirical evidence of FMD in wildlife and their role in the epidemiology and (ii) to identify strategies used or proposed in response to the occurrence of FMD in a wildlife population or at the wildlife‐livestock interface. The information synthesized in this review will aid in the refinement of contingency plans for FMD in Canada, addressing the lack of prevention and control measures aimed at wild animal populations.

## 2. Methods

### 2.1. Development of Research Question and Approach

The broad research question guiding this review is “What is the susceptibility of wildlife species to FMD and the strategies used or proposed in a response to FMD in a wildlife population?”

To comprehensively explore and assess the current body of literature on FMD in wildlife, we conducted a scoping review using the PRISMA scoping review checklist to guide the methodology. Prior to commencing the review, we developed an a priori protocol specifying the review objectives, eligibility criteria, search strategy, study selection process, and data extraction methods. The protocol is provided in the Supporting Information 1; however, it was not prospectively registered or separately published. To achieve the objectives of this review, the following questions were used to develop the search strategy and eligibility criteria: (1) “What international jurisdictions have reported on interventions related to FMD in wildlife?”, (2) “What primary research studies or surveys have been conducted on FMD in wild animal hosts?”, and (3) “What are the surveillance, monitoring, and disease control actions described in the literature?”

### 2.2. Literature Search Strategy

We conducted systematic literature searches between March 28 and April 4, 2025, across five electronic databases: PubMed via NCBI, Web of Science, Agricola via ProQuest, CAB Direct, and Scopus. Studies were eligible to be included if they were published in the year 2000 or later and had a defined target animal population of any domesticated or nondomesticated species that is free‐ranging and does not depend on humans for food or reproduction (“wildlife”). Additionally, the publication type had to fall within one of two categories: (i) primary research, narrative review, or commentary that addressed the role or impact of FMD in target animal populations and/or the control of FMD in target animal populations or (ii) a publication from an international jurisdiction or organization (reports, websites, etc.) that described policies, plans, recorded impact, or interventions regarding the control of FMD in target animal populations. While search terms were in English, we screened records in all languages using an artificial intelligence translator [18]. We tailored search terms and strategies to the requirements and structure of each database (Table S1).

We searched the gray literature between 8 April and 14 May 2025 to capture relevant nonpeer‐reviewed records, especially those from international jurisdictions such as FMD contingency plans. We searched the websites of international jurisdictions and intergovernmental organizations (e.g., Food and Agriculture Organization [FAO], the European Commission for the control of FMD [EuFMD], and the Pan American Center for FMD [PANAFTOSA]) and Google using tailored keyword strings in English similar to those used in the academic database searches (Table S2).

### 2.3. Publication Screening and Selection

We uploaded search results into Covidence systematic review software [19] which automatically compiled and removed duplicates. We manually removed any further duplicates during subsequent screenings. We moved the remaining sources into title and abstract screening, and each was reviewed by one reviewer. If the source title or abstract fulfilled the inclusion criteria, we completed a full‐text review and data extraction. Any uncertainties about inclusion at the abstract screening stage were brought to the group, and a decision was made by consensus.

Separately, we identified gray literature sources in the first 100 Google results for each query and screened all results from relevant international jurisdictions and organizational websites according to the criteria described above. We then uploaded records to Covidence for full‐text review and data extraction as per the peer‐review records.

Two separate reviewers, who were blinded to each other’s evaluation, completed the full‐text screening, using predefined inclusion and exclusion criteria (Table S3). If the reviewers disagreed on the decision of whether to include the publication, it was automatically flagged by the Covidence software, and we then discussed the paper to achieve a consensus ruling.

### 2.4. Data Extraction, Analysis, and Synthesis

We extracted data from each publication into a Microsoft Excel spreadsheet matrix including details of the study and broad thematic categories including species susceptibility, sampling and surveillance, control measures, and specific epidemiological and contextual questions of relevance to wildlife (Table S4). Using the matrix, we used a structured synthesis technique to generate, analyze, and summarize common themes and illustrative examples for each of the topics [20]. Disagreements and questions that arose during data extraction, analysis, and synthesis processes were discussed to achieve consensus. Consistent with the scoping review approach, we did not formally assess study quality or risk of biases because the review aimed to map and characterize the existing literature.

## 3. Results and Discussion

A total of 205 records were included for full data extraction (see Supporting Information for the PRISMA flow diagram).

### 3.1. General Characteristics

Most publications included for full analyses were observational studies (*n* = 67) and reports (*n* = 44) that were published between 2010 and 2016 (*n* = 78), primarily from South Africa (*n* = 33), the United States of America (*n* = 16), and Bulgaria (*n* = 11). The broad thematic categories we explored in this review are wildlife susceptibility to FMDV (*n* = 74), surveillance (*n* = 125), either implemented or recommended, and interventions targeting wildlife, including outbreak prevention and control (*n* = 110).

### 3.2. Susceptibility

Globally, a wide range of mammals are susceptible to FMDV, with infections in particular in ungulate families implicated in disease outbreaks and subsequent transmission. Families of most concern include the *Cervidae*, *Bovidae*, and *Suidae*, but susceptibility in other ungulate families and other species is known and has been extensively reviewed elsewhere (e.g., [12, 15, 21, 22]). Our review focused on the wild and feral animals of particular concern in the Canadian context that have potential range overlap with areas of agricultural production (Table 1).

**Table 1:** Canadian species of concern for potential foot‐and‐mouth disease susceptibility and transmission that have range overlap with agricultural areas.

Family	Scientific name	Common name	Status	Susceptibility to FMDV	Conservation status^a^	Reference
Antilocapridae	*Antilocapra americana*	Pronghorn	Wild	Clinical disease following experimental infections	Not at risk	[23]

Bovidae	*Bison bison bison*	North American plains bison	Wild	Suspected natural infections in captive animals; clinical disease following experimental infections	Threatened	Summarized in [15], [24]
	*Bison bison anthabascae*	North American wood bison	Wild	Likely susceptible	Special concern	—
	*Ovis canadensis*	Bighorn sheep	Wild	Likely susceptible	Not at risk	—
	*Ovis dalli*	Dall/stone sheep	Wild	Likely susceptible	Not at risk	—
	*Oreamnos americanus*	Mountain goat	Wild	Likely susceptible	Not at risk	—

Cervidae	*Odocoileus virginianus*	White‐tailed deer	Wild	Clinical disease following experimental infections	Not at risk	Summarized in [15]
	*Odocoileus hemionus*	Mule deer	Wild	Suspected natural infections in wild animals; clinical disease following experimental infections	Not at risk	Summarized in [15], [25]
	*Cervus canadensis*	Elk	Wild	Clinical disease following experimental infections	Not at risk	[24]
	*Alces alces*	Moose	Wild	Suspected natural infections in wild animals	Not at risk	[26]
	*Rangifer tarandus*	Caribou	Wild	Confirmed natural infections	Threatened or endangered^b^	Summarized in [15]

Suidae	*Sus scrofa*	Boar	Feral	Confirmed natural infections	Invasive	Summarized in [15]

Ursidae	*Ursus americanus*	Black bear	Wild	Likely susceptible	Not at risk	—
	*Ursus arctos*	Brown (grizzly) bear	Wild	Suspected natural infections in captive animals	Special concern	Summarized in [15]

^a^Based on Committee on the Status of Endangered Wildlife in Canada (COSEWIC).

^b^Assessments vary depending on population.

There are 11 native wild species of artiodactyls in Canada that are known or suspected to be susceptible to FMDV. In addition, introduced European boar and feral pigs are also susceptible. Muskox and polar bears are also likely to be susceptible to infection, but their range does not substantially overlap with areas of agricultural production. Finally, in species for which susceptibility data are not available (bighorn sheep, stone/Dall’s sheep, and mountain goats), it can be inferred that these are highly likely to be susceptible based on data in closely related species and the broad host range of FMDV. However, understanding the expected infection dynamics in each of the Canadian species that may have a role in FMD transmission is necessary for response planning. Effective preparedness must also include complete and accessible data pertaining to the biology of susceptible species, including their population densities and geographical distribution, among other factors [12, 17].

Experimental infections of wildlife native to Canada are useful to understand the clinical signs, lesions, and clinical course of infection in these animals. For instance, an experimental infection study confirmed that pronghorn are susceptible to infection, develop severe characteristic lesions, and can spread the virus via intraspecific transmission and to cattle in a laboratory setting [23]. However, extrapolating this information to the potential role of these species in an outbreak is challenged by the range of clinical signs and lack of data regarding transmission in natural settings. In general, there is a range of clinical signs in wildlife species; those with limited clinical signs could go undetected and potentially transmit the virus over great geographical distances.

Sustained infection in wildlife requires large populations with ongoing recruitment or reservoir hosts [27]. Modeling studies have concluded that FMDV infection in wild or feral pig and deer populations is usually self‐limiting in the absence of livestock transmission [28, 29] with possible exceptions in high‐density mixed‐species populations [30, 31] or geographically extensive and connected patches [32]. Seasonal breeding in Canadian wildlife limits recruitment of susceptible hosts; however, environmental persistence could offset these population factors by providing a source of infection [27].

The ability of native Canadian wildlife species to act as reservoir species, as occurs in African buffalo, is unknown. But the possibility that species such as bison, wild sheep, and goats could become long‐term carriers is worthy of consideration in response planning and could be investigated by experimental infections. Historical experimental infection data in white‐tailed deer indicate the potential for persistent carrier states for up to 11 weeks [12]. Except for buffalo, the presence of carrier states in wildlife is species‐specific, variable, and of questionable transmission risk [12].

Beyond their potential role in disease transmission, wildlife may be directly impacted by FMDV infection and reactive control methods, such as culling and fencing. An outbreak may have substantial biodiversity and conservation impacts. In the Canadian context, wildlife species that are likely susceptible to FMD and have conservation concerns include caribou, bison, and grizzly bears (Table 1) [33].

In the event of a Canadian FMD outbreak, deer, bison, and pronghorn would be expected to experience high morbidity and mortality rates if the virus was introduced into these populations and the right ecological factors were met to support sustained transmission (e.g., season and sufficient number of new susceptible hosts) [23–25]. However, these assumptions are mainly derived from experimental infections that may not reflect a field outbreak scenario. The exception is the possible 1924 outbreak of FMD in mule deer in California that resulted in massive culling and the identification of suggestive lesions that were not laboratory‐confirmed [12]. Effects on infected individuals may include direct disease from the virus, increased susceptibility to secondary infections, and decreased overall health impacting fitness and reproduction. Animals with lameness from FMD lesions may be preferentially targeted by predators.

The role of wildlife ecology in FMD transmission is complex. Ecological factors that affect wild animals’ ability to transmit infection to and between domestic species include season, population densities, and behavioral and landscape features. The demographics of infected animals may also be important; for instance, males typically have larger home ranges and greater opportunities for contact via conspecific fights and mating behaviors, which may promote disease spread, as is seen with chronic wasting disease transmission in mule deer [34]. Species such as mule deer and pronghorn overlap with agricultural areas and can travel significant geographical distances, with the potential to spread FMD between disparate farms (Figure 1); however, clinical disease including lameness may somewhat limit this potential spread [23, 25].

**Figure 1:**
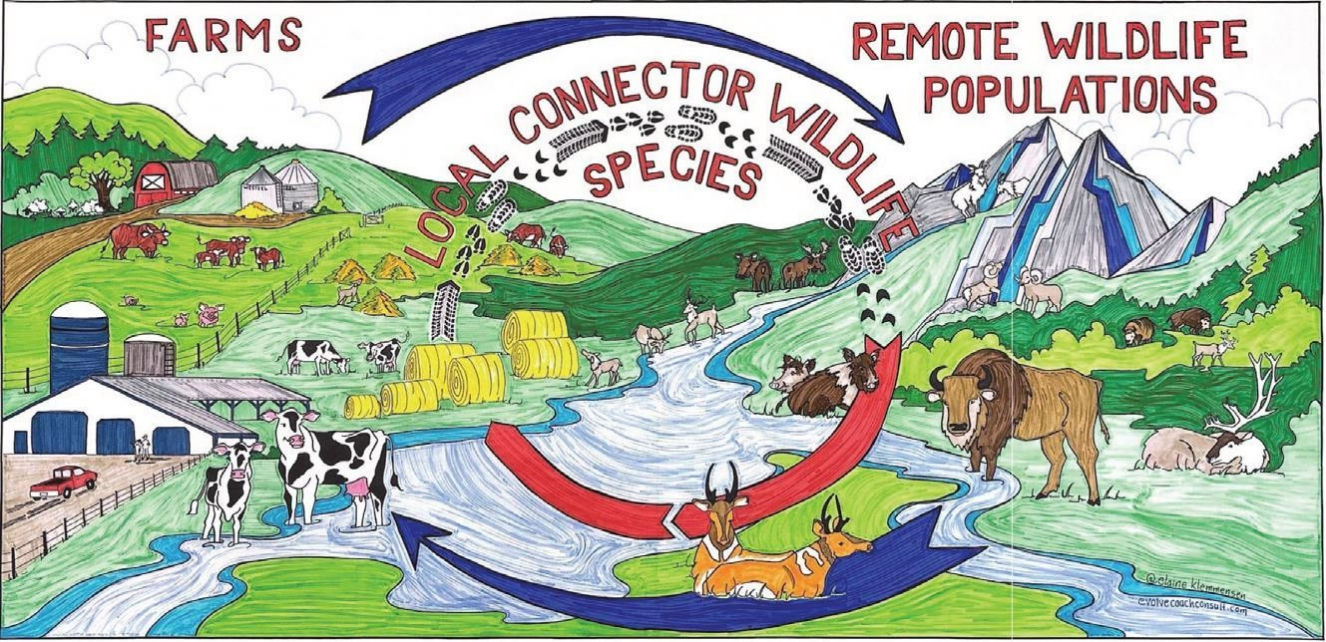
Potential foot‐and‐mouth disease transmission pathways at livestock‐wildlife interfaces in Canada. Local connector wildlife species include those with frequent and sustained overlap with livestock farms. These species may contribute to long‐term disease maintenance and facilitate long‐range transmission to remote wildlife populations unlikely to contact livestock directly. Shared environments such as water sources and feed storage areas represent key seasonally dependent points of potential contact that may facilitate virus transmission. Anthropogenic fomites, including footwear and vehicles, may also contribute to virus spread. Illustration by Dr. Elaine Klemmensen, Evolve Leadership Coaching and Consulting.

The livestock‐wildlife interface includes shared habitats and resources (e.g., water and food) as sites of potential virus transmission, in addition to direct contact (Figure 1). Water sources are the main sites of disease spread between livestock and wildlife, and this is exacerbated by dry seasons where limited water availability concentrates animals to specific locations [35–39]. This suggests that with periods of drought expected to increase in Canada with climate change, this may increase the potential risk [40].

### 3.3. Monitoring and Surveillance

We defined surveillance and monitoring of FMD in wildlife broadly to include both the conventional definition of surveillance as ongoing collection of animal health data for control purposes, as well as observational studies that involve the collection of samples or specimens aimed at detecting or monitoring FMD [41]. Ninety publications fit this definition of surveillance.

Most records that reported surveillance or monitoring activities described observational studies conducted within defined timeframes. Many records were surveys designed to detect FMDV or evidence of prior exposure, most commonly through serological surveys. Some records reported wildlife sampling during or following outbreaks to assess or characterize wildlife exposure to FMDV [42–44] or to identify the outbreak source [37, 45]. Passive surveillance has likewise proven valuable in outbreak situations to identify the disease in found‐dead wild animals [44]. In FMD‐free settings, observational studies of FMD were often embedded within broader wildlife health investigations [46–48]. Most publications described a risk‐based (*n* = 58) or convenience‐based (*n* = 35) sampling strategy, and no records described population‐based sampling. Studies that examined FMDV transmission within wildlife populations used modeling methodologies. Serology was the most common type of diagnostic test performed (*n* = 58), with nonstructural protein ELISA (NSP ELISA) as the most common serological test (*n* = 30). Pathogen detection was most often done using RT‐PCR (*n* = 41), followed by virus isolation (*n* = 33). Specimens collected for virus detection included esophageal‐pharyngeal/oropharyngeal samples (*n* = 22), followed by epithelial tissues, organs (*n* = 11 each), and saliva (*n* = 10).

The operational plans or strategic frameworks (*n* = 9) that address FMD surveillance in wildlife during an outbreak typically refer to it in broad terms, often as opportunistic sampling of hunter‐harvested or found‐dead animals. Some plans offered high‐level guidance on demographic or epidemiological considerations for targeted, risk‐based wildlife surveillance [49, 50], while others explicitly called for coordination with wildlife agencies [51, 52]. The European Union [53] also includes provisions for wildlife surveillance for transmissible animal diseases within relevant legislation. A prioritization exercise in Europe ranked FMDV second among pathogens to be targeted in wildlife surveillance programs [54]. Contingency plans often recommend the establishment of specialized wildlife expert groups comprising of epidemiologists, biologists, wildlife veterinarians, and other scientific experts to assess and recommend actions in response to suspected or confirmed FMD outbreaks in wild animal population [49, 50, 55]. Plans also emphasize formal coordination with national wildlife and environmental agencies, research institutes, and nongovernmental organizations, to manage outbreaks effectively and strengthen surveillance coverage and data quality [52, 56].

Challenges to FMDV surveillance in wildlife relate to the known drawbacks of different surveillance strategies including but not limited to the reliance of passive surveillance on public reporting, the stoic behavior of ungulate species; the fact that active surveillance is time‐consuming, expensive, and invasive; and that hunter‐harvested surveillance is seasonally limited, which may delay detection [45, 57, 58]. The importance of validated noninvasive sampling techniques was highlighted as a solution to some of these challenges [58, 59].

Our results support the need for ongoing passive wildlife disease surveillance. It is possible in the event of an introduction that infection may first happen in susceptible wild animals. The Canadian Wildlife Health Cooperative (CWHC), a pan‐Canadian partnership between the country’s five veterinary schools and the British Columbia provincial diagnostic laboratory, oversees wildlife health surveillance activities in this country. Ongoing challenges to CWHC’s work include vast geographical areas with sparse human population density, inconsistent funding models with wildlife falling between and among various jurisdictions, and a bias towards charismatic megafauna [60]. There needs to be ongoing recognition of the critical importance of wildlife disease surveillance activities and the potential role this work may have in detecting an incursion of FMDV. In addition to the continued strengthening of Canada’s wildlife surveillance system, our results strongly support the incorporation of a risk‐based approach to active surveillance of wildlife in FMD contingency planning, developed in partnership with wildlife health experts, agencies, and stakeholders. Active surveillance strategies should be developed to increase the probability of detecting spillover to wildlife in the face of an FMD outbreak while addressing logistical and epidemiological challenges. Ideally, these strategies would be informed by risk assessments and incorporate livestock‐wildlife interfaces (e.g., geographical areas and species), range overlaps, and migratory species routes along with other relevant factors. They may also incorporate human activities that enable opportunistic sampling, such as hunting or feral pig eradication efforts. General considerations when developing FMD surveillance in wildlife can be found in Figure 2.

**Figure 2:**
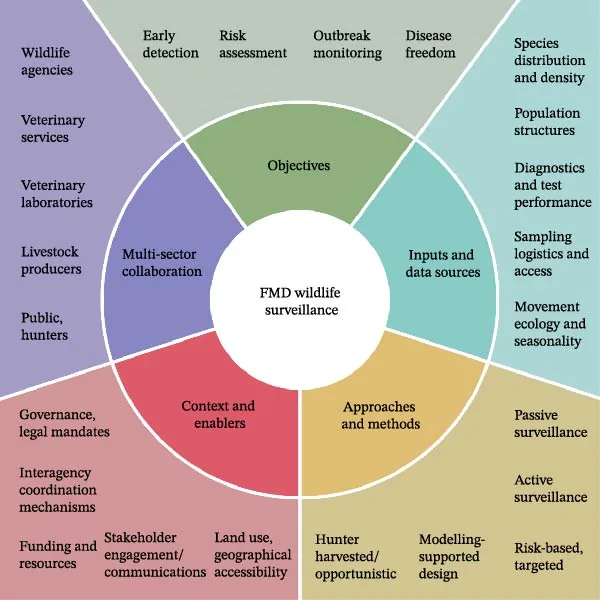
Considerations when designing surveillance plans for FMD in wildlife.

### 3.4. Control and Interventions in Wildlife Populations

Publications that described control measures in wildlife populations consisted of observational studies that made recommendations based on their results, contingency and operational plans, and outbreak response reports. Contingency plans and strategies identified in the gray literature that included actions related to wildlife generally advocated for flexible, case‐by‐case approaches, emphasizing coordination between veterinary authorities and wildlife agencies. Control strategies targeting wildlife are summarized in Table 2. The most frequently discussed control measures among these were wildlife population management (*n* = 44), vaccination of livestock at the wildlife‐livestock interface (*n* = 41), and use of large‐scale fencing (*n* = 49), though the latter two were specific to southern Africa where African buffalo serve as a reservoir species (see Section 4 for more details).

**Table 2:** Summary of proposed and implemented interventions to control foot‐and‐mouth disease in wildlife, derived from a scoping literature review.

Intervention	Notes	Reported effectiveness	Constraints	Example reference
Enhanced biosecurity (e.g., reducing wildlife attractants on farms)	Proposed in contingency plans	Unknown	Uptake/adherence by producers	[36]
Fencing	Primarily implemented in Southern Africa	High (transmission barrier)	Costly, environmental fragmentation	[61]
No action	Implemented in multiple outbreaks	Viable if self‐limiting	Risk to vulnerable species and establishment in maintenance hosts, may prolong outbreak	[16]
Vaccination (livestock)	Interface zones	High (ring vaccination)	Requires strain match	[62]
Vaccination (wildlife)	No recorded examples	Unknown	Logistically and epidemiologically challenging	[49]
Wildlife culling	Risk based, clinical disease	Low/short‐term	Public opposition, ecological impact	[63]
Zoning of wildlife‐infected area (restrictions on wildlife products and domestic populations)	Proposed in contingency plans	Unknown	Evidence base could be difficult to gather	[49]

During active FMD outbreaks, the primary control strategies reported involved various forms of depopulation. For instance, culling of susceptible wild goats and deer in common grazing areas was completed during the 2001 outbreak in Great Britain, the Republic of Ireland, and the Netherlands [22, 64]. In the outbreak in Bulgaria in 2011, only wild boar showing clinical signs were culled [65], with recommendations to continue trapping, testing, and culling positive animals (identified by rapid tests) as a precautionary measure [66]. Other control measures discussed in response to outbreaks were to restrict the movement of hunted wild animals outside of affected regions [67] and ban hunting in these regions [68].

These responses were consistent with recommendations made in contingency or operational plans we reviewed, which propose the establishment of “Wildlife Infected Zones” to control the movement of animals and animal products [49, 69, 70] and culling of wildlife when deemed necessary [49, 56, 69]. Conversely, some contingency plans stated that wildlife culling was not feasible nor recommended [50, 71]. Commonly, these plans called for the creation of a working group of experts if FMD was detected in wildlife (see above), to determine the most appropriate control actions for the specific population.

Several modeling studies (*n* = 17) explored control methods and their efficacy, primarily concerning depopulation. In white‐tailed deer populations, the efficacy of culling was found to be affected by landscape type, culling method, and population density [30, 63]. In wild boar, prophylactic management of population numbers was found to reduce outbreak risk, but culling after detection of the virus was unlikely to be effective in reducing outbreak size, especially if wild boar had frequent livestock contact [28, 31]. Other measures, such as reducing attractants on farms, had a significant effect on disease spread [72], while utilizing fencing to separate wildlife and livestock reduced transmission risk significantly in South Africa [38, 73, 74].

Overall, culling of wildlife is generally not feasible or supported by the public [17, 58, 71, 75–77]. There are often real or perceived environmental harms [50, 56, 58]. Culling is only recommended in narrowly defined situations where population management or targeted removal of infected animals can reduce transmission risk (i.e., certain ecoregions, population dynamics, stage of an outbreak, or prophylactic population control) [30, 32, 58, 63, 78].

Enhanced biosecurity other than fencing may prevent contact between livestock and wildlife. This includes reducing wildlife attractants on farms such as livestock feed, storage, handling sites, waterers, and mineral licks and managing possible mechanical vectors such as rodents and flies [50, 52, 53, 69, 76]. Furthermore, there is the possibility that certain wildlife species may act as mechanical vectors for FMDV, although the role of scavengers (e.g., foxes, seagulls, and vultures), rodents, and synanthropic species that congregate in dense populations (e.g., starlings and house sparrows) in the transmission of FMD remains poorly understood and largely unquantified [50]. Despite this limited evidence, vector control is recommended within enhanced biosecurity protocols as a precautionary measure [52, 56].

Fencing reduces transmission but is costly and ecologically disruptive. This is a primary method of reducing wildlife‐livestock transmission in Southern Africa, though their cost and potential negative environmental and tourism impacts are acknowledged. Other than the Southern African region, this method was proposed in one other contingency plan as an option to separate livestock and wildlife in high‐risk zones [49, 56, 79, 80]. If implemented, fencing should use the landscape’s natural barriers as part of zoning and fence erection to reduce costs and environmental impacts [81]. Fence heights must act as an effective barrier to all species effectively reduce transmission [82].

Contact rates between wildlife and livestock vary seasonally, driven by changes in wildlife behavior and livestock management, including access to feed and water, grazing patterns, and breeding or calving seasons [83, 84]. This suggests that effective FMD control measures must incorporate these seasonal patterns, especially in highly seasonal climates, such as Canada. Baiting animals for capture, culling, or to draw them away from boundaries is explicitly advised against due to increased transmission risk with animal congregation [49].

While vaccination of wildlife was not a strategy we identified in the literature review, considerations relevant to Canada and other nonendemic regions include direct disease and control risks to vulnerable species, logistical and financial challenges in vaccine delivery, and effective coverage and efficacy.

In some contexts, taking a noninterventionist approach may have negligible consequences. Deciding whether to implement control measures for FMD in wildlife populations involves two epidemiological considerations: the role of wildlife in sustaining transmission and the potential conservation impacts on vulnerable species.

Overall, existing modeling and empirical evidence suggest that FMDV is expected to fade out in most wildlife populations in the absence of continual reintroduction from domestic livestock. Consequently, if FMDV is unlikely to persist in wildlife, control efforts could be focused solely on eliminating the virus in domestic livestock. This approach was effectively adopted for wild animal populations during FMD outbreaks in the United Kingdom in 2001 and Bulgaria in 2010 [16, 27] and is mentioned as a potentially viable option in at least one contingency plan [52]. This body of international work highlights the need for Canada to build its own evidence base related to the likelihood of viral persistence and transmission dynamics in its native wild populations, similar to studies conducted in other ecosystems.

Conversely, a nonintervention strategy may pose significant risks to vulnerable species. Allowing FMDV to transmit from infected livestock to wildlife can have severe consequences for their survival, as evidenced by outbreaks that caused high mortality in mountain gazelles in Israel and saiga antelope in Kazakhstan [12]. Many North American ungulates are susceptible to FMD, but little is known about direct and indirect mortality rates in these populations. Therefore, risk assessments are critical to developing targeted, evidence‐based strategies to mitigate the impact of FMD outbreak on both domestic and vulnerable wild species. At least one framework has been developed for assessing spillover risk to migratory species [85].

While this review did not focus on the economics of FMD control strategies, none of the publications we retrieved reported on cost/benefit analyses of wildlife interventions. Regardless, both economic and conservation considerations should be weighed when formulating FMD interventions targeting wildlife, including the possible negative impacts on survival and biodiversity.

Overall, recommendations for the management of FMD in wildlife highlight the importance of engaging wildlife agencies in outbreak response and preparedness planning, delineating wildlife infected zones to regulate animal movement and hunting activities, and strengthening biosecurity measures at the livestock–wildlife interface. The culling of wildlife is generally considered infeasible or undesirable and is recommended only under exceptional circumstances where epidemiologically justified. There also needs to be consideration for animal welfare in control plans to minimize distress and harm to animals. Control strategies for FMDV must balance epidemiological effectiveness with ecological and social acceptability.

### 3.5. FMD in Sub‐Saharan African Wildlife—Lessons From an Endemic Zone

The wildlife‐livestock interface in sub‐Saharan Africa, where FMD is endemic, is the most extensively studied system of its kind. African buffalo (*Syncerus caffer*) are highly susceptible to infection and are the only confirmed wildlife maintenance host, though other species can sustain persistent infections [12]. Juveniles often become infected as maternal antibodies wane and may transmit the virus [86]. Virus circulation is heterogeneous, with strain differences between buffalo and cattle populations and evidence of interspecific transmission [87–89].

Surveillance focuses on buffalo reservoir populations within official zoning systems. In Namibia, buffalo are generally excluded from the FMD‐free Namibian Export Region (NER), except in one national park where representative animals are serologically tested biennially; any stray buffalo in protection or free zones are culled [90]. In South Africa, designated endemic zones (e.g., Kruger National Park) are separated from FMD‐free areas by protection zones that may have livestock vaccination and active livestock surveillance. Buffalo movements within FMD‐free zones require negative test results and veterinary permits [91].

Because FMD is endemic in some buffalo populations, southern African control strategies focus on reducing transmission between infected and noninfected herds and limiting buffalo–livestock contact. Control relies on zoning and large‐scale fencing, which may effectively reduce transmission [73, 92]. Fencing is costly, prone to damage, and is less effective against species such as impala, which can bypass barriers and act as intermediate carriers [37, 38, 92–95]. It also negatively impacts wildlife populations by restricting movements, fragmenting habitats, and reducing genetic diversity [81, 96–98]. Alternatives include development of commercial livestock areas with natural barriers, developing commodity trade, and community involvement [81, 83, 97, 99].

Livestock vaccination along infected zone boundaries remains a cornerstone of control, requiring frequent doses and antigenic matching to circulating wildlife strains [100, 101]. Other approaches include ring vaccination around endemic buffalo populations, targeted removal of carrier animals, creating disease‐free buffalo herds, and managing shared resources such as forage and water to reduce wildlife‐livestock contact [27, 84]. The strategies in southern Africa are built around long‐term management of an endemic buffalo reservoir. Therefore, they offer limited direct guidance for outbreak response in nonendemic regions. In these regions, principles such as restricting wildlife‐livestock contact and rapid spillover detection are more relevant than reservoir‐focused control measures.

### 3.6. Strengths and Limitations

In keeping with established scoping review methodology, we did not conduct a formal assessment of study quality or risk of bias. The evidence base for this review included a broad range of sources, ranging from observation and modeling studies, surveillance reports, and gray literature that varied widely in their methodological rigor and certainty. Consequently, the results should be interpreted as a characterization and synthesis of the available evidence and identification of knowledge gaps, rather than a formal evaluation of evidence confidence.

## 4. Conclusions

Research on FMD in wildlife has primarily been in relation to livestock and has focused on observational studies, infection trials, and modeling studies. Less commonly, some researchers have explored the impact the disease may have on susceptible wild populations and the conservation implications for vulnerable species. This review confirmed that FMDV is known to affect a wide range of mammals, with at least 13 native Canadian wild animal species, as well as introduced feral pigs, that are known or highly likely to be susceptible to FMD. Experimental infections, such as those conducted on deer and pronghorn, confirm susceptibility and transmission capability in a laboratory setting. However, the dynamics and impact on wild animals in case of a natural outbreak in Canada remain unknown. Population data including densities and geographic distribution of susceptible wild species, a better understanding of infection dynamics, and the expected impact on vulnerable wild populations are key priorities for developing evidence‐informed risk‐based mitigation strategies.

Modeling studies and empirical data in other jurisdictions such as the United States, Australia, and Europe suggest that FMDV infection in wild or feral pig and deer populations is generally self‐limiting in the absence of livestock transmission. This is due to insufficient population sizes and/or densities and the typically short infectious period in these species, suggesting that outbreaks would not persist without reintroduction. This contrasts sharply with regions like southern Africa, where the African buffalo serves as a recognized maintenance host, necessitating comprehensive surveillance and zoning strategies. In the Canadian context, data are lacking on the potential of certain wildlife species, including the North American bison, which belongs to the Bovidae family like the African buffalo, to act as maintenance hosts.

Modeling studies and risk assessments play a vital role in FMD‐free countries by informing surveillance design and plans, yet this review revealed a notable gap in these resources for Canada. Passive surveillance of found‐dead wild animals can be valuable in outbreak situations in naïve populations. However, Canada faces challenges in passive surveillance due to vast geographical areas, inconsistent funding, and sparse population density [60, 102]. This review underscores the need for long‐term support for passive surveillance capacity (e.g., CWHC), animal health networks, and coordinating platforms such as Animal Health Canada (AHC), which provides critical situational awareness and enables rapid activation of expert working groups and coordinated action when an outbreak occurs, capacities that cannot be built during an emergency.

The most frequently discussed prevention and control interventions were wildlife population management (culling/depopulation), fencing, and livestock vaccination at the wildlife‐livestock interface. Contingency plans recommend establishing wild animal infected zones (WAIZs) to restrict movement and hunting if FMD is detected in wildlife. Moreover, the culling of wildlife is generally considered infeasible, often environmentally harmful, and is recommended only under narrowly defined, epidemiologically justified circumstances. In some cases, a “no action” strategy for wildlife, coupled with containment measures, can be considered, particularly if evidence suggests that the virus is self‐limiting in the affected wild population. Culling of native wild animals is neither viable nor justified in most Canadian contexts; instead, enhanced biosecurity at livestock–wildlife interfaces, strategic zoning, and proactive communication with wildlife management partners offer more appropriate tools for mitigating transmission risk.

Historical challenges of disease and population control in Canadian wildlife such as brucellosis and bovine tuberculosis in bison within Wood Buffalo National Park, the establishment of feral pig/wild boar populations, and the unremitting spread of chronic wasting disease serve as cautionary examples to underscore that proactive planning for FMD must account for the inherent difficulties of disease control in Canadian wildlife [34, 103, 104]. Ecological conditions, wildlife population dynamics, and livestock management practices vary substantially between countries, and findings from other jurisdictions, including endemic regions, should inform—rather than be directly extrapolated to—the context in nonendemic countries. Preparedness and contingency planning for FMD should explicitly incorporate wildlife considerations, including mechanisms for data sharing, risk assessments, objectives and strategies, and decision‐making frameworks for wildlife surveillance and control. Future research should include experimental infections to characterize the susceptibility of wildlife species for which data are lacking and evaluate the potential for the establishment of maintenance hosts (e.g., bison). Other priorities include modeling studies to estimate the likelihood of FMDV persistence in key Canadian species and ecological systems, targeted studies to validate surveillance tools and sampling strategies, and the integration of genomic epidemiology into surveillance programs to improve knowledge of transmission dynamics at the wildlife‐livestock interface. Preparedness for FMD in Canada and other countries in which FMD is nonendemic must integrate livestock, wildlife, and environmental dimensions to ensure a coordinated and scientifically defensible response to this high‐impact transboundary disease.

## Funding

Funding to support Erin N. Casper was provided by the Summer Undergraduate Research Experience program through the University of Calgary Faculty of Veterinary Medicine. Emma G. Gardner and Murray Gillies were funded by the Agri Assurance Program under the Sustainable Canadian Agricultural Partnership, a federal‐provincial‐territorial initiative (Grant SCAP‐AAPN‐PAAA‐1199).

## Disclosure

The authors declare that this manuscript is original, has not been published before, and is not currently being considered for publication elsewhere. All authors have approved the manuscript and agree with its submission.

## Conflicts of Interest

The authors declare no conflicts of interest.

## Supporting Information

Additional supporting information can be found online in the Supporting Information section.

## Supporting information


**Supporting Information** Supporting information for this article is available online, including the a priori review protocol used to guide this scoping review and the PRISMA‐ScR flow diagram summarizing the study identification, screening, and inclusion process.

## Data Availability

The data that support the findings of this study are available from the corresponding author upon reasonable request.

## References

[1] Animal Health Canada , Foot and Mouth Disease, 2025, Accessed November 4, 2025 https://animalhealthcanada.ca/foot-and-mouth-disease.

[2] Adams M. , Lefkowitz E. , King A. , and Carstens E. , Recently Agreed Changes to the International Code of Virus Classification and Nomenclature, Archives of Virology. (2013) 158, no. 12, 2633–2639. 10.1007/s00705-013-1749-9 23836393

[3] Grubman M. J. and Baxt B. , Foot-and-Mouth Disease, Clinical Microbiology Reviews. (2004) 17, no. 2, 465–493, 10.1128/CMR.17.2.465-493.2004. PMC387408 15084510

[4] Kitching R. P. and Alexandersen S. , Clinical Variation in Foot and Mouth Disease: Pigs, Revue Scientifique Et Technique-Office International Des Epizooties. (2002) 21, no. 3, 513–518, 10.20506/rst.21.3.1367. 12523692

[5] Kitching R. P. , Clinical Variation in Foot and Mouth Disease: Cattle, Revue Scientifique Et Technique-Office International Des Epizooties. (2002) 21, no. 3, 499–502, 10.20506/rst.21.3.1343. 12523690

[6] Knight-Jones T. J. and Rushton J. , The Economic Impacts of Foot and Mouth Disease–What are They, How Big are They and Where do They Occur?, Preventive Veterinary Medicine. (2013) 112, 161–173. 10.1016/j.prevetmed.2013.07.013 PMC3989032 23958457

[7] Government of Canada , Canadian Food Inspection Agency , and Fact Sheet , Foot and Mouth Disease, 2025, Accessed November 4 https://inspection.canada.ca/en/animal-health/terrestrial-animals/diseases/reportable/foot-and-mouth-disease/fact-sheet.

[8] Cairns A. , Tolhurst T. , and Poon K. , et al.The Economic Impact of a Foot-and-Mouth Disease Outbreak for Ontario’s Beef Sector, Canadian Journal of Agricultural Economics/Revue Canadienne D’agroeconomie. (2017) 65, no. 1, 159–183, 10.1111/cjag.12108.

[9] Tozer P. R. , Marsh T. L. , and Perevodchikov E. V. , Economic Welfare Impacts of Foot-and-Mouth Disease in the Canadian Beef Cattle Sector, Canadian Journal of Agricultural Economics/Revue Canadienne D’agroeconomie. (2015) 63, no. 2, 163–184, 10.1111/cjag.12041.

[10] Mepham B. , Foot and Mouth Disease and British Agriculture: Ethics in a Crisis, Journal of Agricultural and Environmental Ethics. (2001) 14, no. 3, 339–347, 10.1023/A:1012298010842.

[11] Whiting T. L. , Foreign Animal Disease Outbreaks, the Animal Welfare Implications for Canada: Risks Apparent From International Experience, The Canadian Veterinary Journal. (2003) 44, no. 10, 805–815. PMC340296 14601676

[12] Weaver G. V. , Domenech J. , Thiermann A. R. , and Karesh W. B. , Foot and Mouth Disease: A Look From the Wild Side, Journal of Wildlife Diseases. (2013) 49, no. 4, 759–785, 10.7589/2012-11-276. 24502706

[13] Wiethoelter A. K. , Beltrán-Alcrudo D. , Kock R. , and Mor S. M. , Global Trends in Infectious Diseases at the Wildlife–Livestock Interface, Proceedings of the National Academy of Sciences. (2015) 112, no. 31, 9662–9667, 10.1073/pnas.1422741112. PMC4534210 26195733

[14] Gortázar D. C. , Vicente J. , and Höfle U. , Mitigating Disease Transmission Risk at the Wildlife–Livestock Interface to Facilitate Safe Trade, 2025, Accessed September 24, 2026, World Organisation for Animal Health (WOAH), Paris, France, https://www.woah.org/app/uploads/2025/12/2025-guidelines-wildlife-livestock-interface-en.pdf, 10.20506/woah.3649.

[15] Rahman A.-U. , Dhama K. , Ali Q. , Raza M. A. , Chaudhry U. , and Shabbir M. Z. , Foot and Mouth Disease in a Wide Range of Wild Hosts: A Potential Constraint in Disease Control Efforts Worldwide Particularly in Disease-Endemic Settings, Acta Tropica. (2020) 210, 10.1016/j.actatropica.2020.105567, 105567. 32504589

[16] Gortázar C. , Barroso P. , Nova R. , and Cáceres G. , The Role of Wildlife in the Epidemiology and Control of Foot-and-Mouth-Disease and Similar Transboundary (FAST) Animal Diseases: A Review, Transboundary and Emerging Diseases. (2022) 69, no. 5, 2462–2473, 10.1111/tbed.14235. 34268873

[17] Leighton F. A. , Foreign Animal Diseases and Canadian Wildlife: Reasons for Concern and the Elements of Preparedness, Canadian Veterinary Journal. (2002) 43, 265–267. PMC339232 11963659

[18] DeepL Translator , 2025, DeepL SE, Cologne, Germany, https://www.deepl.com/translator.

[19] Covidence Systematic Review Software , Melbourne, Australia, https://www.covidence.org.

[20] Garrard J. , Health Sciences Literature Review Made Easy: The Matrix Method, 2022, 6th edition, Jones & Bartlett, Burlington, MA.

[21] Thomson G. R. , Vosloo W. , and Bastos A. D. S. , Foot and Mouth Disease in Wildlife, Virus Research. (2003) 91, no. 1, 145–161, 10.1016/S0168-1702(02)00263-0. 12527441

[22] Pinto A. A. , Foot-and-Mouth Disease in Tropical Wildlife, Annals of the New York Academy of Sciences. (2004) 1026, no. 1, 65–72, 10.1196/annals.1307.008. 15604471

[23] Rhyan J. , McCollum M. , and Gidlewski T. , et al.Foot-and-Mouth Disease in a Small Sample of Experimentally Infected Pronghorn (*Antilocapra americana*), Journal of Wildlife Diseases. (2016) 52, no. 4, 862–873, 10.7589/2015-11-312. 27525593

[24] Rhyan J. , Deng M. , and Wang H. , et al.Foot-and-Mouth Disease in North American Bison (*Bison bison*) and Elk (*Cervus elaphus* Nelsoni): Susceptibility, Intra- and Interspecies Transmission, Clinical Signs, and Lesions, Journal of Wildlife Diseases. (2008) 44, no. 2, 269–279, 10.7589/0090-3558-44.2.269. 18436660

[25] Rhyan J. , McCollum M. , and Gidlewski T. , et al.Foot-and-Mouth Disease in Experimentally Infected Mule Deer (*Odocoileus hemionus*), Journal of Wildlife Diseases. (2020) 56, no. 1, 93–104, 10.7589/2019-03-059. 31329525

[26] Magnusson H. , Ein Fall Von Maul-Und Klauenseuche Beim Elch, Deutsche Tierärztliche Wochenschrift. (1939) 47, 509–511.

[27] Anderson E. , Foot-and-Mouth Disease Control Programmes: Factors That Influence the Strategy for Wildlife, 2003, Editions Scientifiques Et Medicales Elsevier, 145–151.

[28] Pepin K. M. and VerCauteren K. C. , Disease-Emergence Dynamics and Control in a Socially-Structured Wildlife Species, Scientific Reports. (2016) 6, no. 1, 10.1038/srep25150, 25150. PMC4844964 27114031

[29] Dhollander S. , Belsham G. , and Lange M. , et al.Assessing the Potential Spread and Maintenance of Foot-and-Mouth Disease Virus Infection in Wild Ungulates: General Principles and Application to a Specific Scenario in Thrace, Transboundary and Emerging Diseases. (2016) 63, no. 2, 165–174, 10.1111/tbed.12240. 24903641

[30] Ward M. P. , Laffan S. W. , and Highfield L. D. , Modelling Spread of Foot-and-Mouth Disease in Wild White-Tailed Deer and Feral Pig Populations Using a Geographic-Automata Model and Animal Distributions, Preventive Veterinary Medicine. (2009) 91, no. 1, 55–63, 10.1016/j.prevetmed.2009.05.005. 19515439

[31] Ward M. P. , Garner M. G. , and Cowled B. D. , Modelling Foot-and-Mouth Disease Transmission in a Wild Pig-Domestic Cattle Ecosystem, Australian Veterinary Journal. (2015) 93, no. 1-2, 4–12, 10.1111/avj.12278. 25622702

[32] Croft S. , Aegerter J. N. , Massei G. , Smith G. C. , and Woźniakowski G. , The Risk of Foot-and-Mouth Disease Becoming Endemic in a Wildlife Host is Driven by Spatial Extent Rather Than Density, PLoS One. (2019) 14, no. 6, 10.1371/journal.pone.0218898. PMC6594678 31242228

[33] Government of Canada , Species at Risk Public Registry, 2025, Accessed November 10, 2025 https://www.canada.ca/en/environment-climate-change/services/species-risk-public-registry.html.

[34] Escobar L. E. , Pritzkow S. , and Winter S. N. , et al.The Ecology of Chronic Wasting Disease in Wildlife, Biological Reviews. (2020) 95, no. 2, 393–408, 10.1111/brv.12568. PMC7085120 31750623

[35] Atuman Y. , Kudi C. , and Abdu P. , et al.Seroprevalence of Foot and Mouth Disease Virus Infection in Some Wildlife and Cattle in Bauchi State, Nigeria, Veterinary Medicine International. (2020) 2020, 1–8, 10.1155/2020/3642793, 3642793. PMC7104331 32257095

[36] Cooper S. M. , Scott H. M. , De La Garza G. R. , Deck A. L. , and Cathey J. C. , Distribution and Interspecies Contact of Feral Swine and Cattle on Rangeland in South Texas: Implications for Disease Transmission, Journal of Wildlife Diseases. (2010) 46, no. 1, 152–164, 10.7589/0090-3558-46.1.152. 20090028

[37] Hargreaves S. , Foggin C. , and Anderson E. , et al.An Investigation Into the Source and Spread of Foot and Mouth Disease Virus From a Wildlife Conservancy in Zimbabwe, Revue Scientifique Et Technique De l’oie. (2004) 23, no. 3, 783–790, 10.20506/rst.23.3.1519. 15861873

[38] Dion E. and Lambin E. F. , Scenarios of Transmission Risk of Foot-and-Mouth With Climatic, Social and Landscape Changes in Southern Africa, Applied Geography. (2012) 35, no. 1-2, 32–42, 10.1016/j.apgeog.2012.05.001.

[39] DiNardo A. , Libeau G. , and Chardonnet B. , et al.Serological Profile of Foot-and-Mouth Disease in Wildlife Populations of West and Central Africa With Special Reference to *Syncerus caffer* Subspecies, Veterinary Research. (2015) 46, no. 1, 10.1186/s13567-015-0213-0, 77. PMC4495843 26156024

[40] Yusa A. , Berry P. , and Cheng J. J. , et al.Climate Change, Drought and Human Health in Canada, International Journal of Environmental Research and Public Health. (2015) 12, no. 7, 8359–8412, 10.3390/ijerph120708359. PMC4515727 26193300

[41] Thrusfield M. , Veterinary Epidemiology, 2018, John Wiley & Sons.

[42] Letshwenyo M. , Mapitse N. , and Hyera J. M. K. , Foot-and-Mouth Disease in a Kudu (*Tragelaphus strepsiceros*) in Botswana, Veterinary Record. (2006) 159, no. 8, 252–253, 10.1136/vr.159.8.252. 16921016

[43] Nyamsuren D. , Joly D. O. , and Enkhtuvshin S. , et al.Exposure of Mongolian Gazelles (*Procapra gutturosa*) to Foot and Mouth Disease Virus, Journal of Wildlife Diseases. (2006) 42, no. 1, 154–158, 10.7589/0090-3558-42.1.154. 16699158

[44] Etinger M. , Pozzi P. , Bellaiche M. , Hamed F. , and Tov B. , Review of the Occurrence of FMD in Israel and a Clinical Description of the Outbreak of the Disease in 2021, Israel Journal of Veterinary Medicine. (2022) 77, 151–160.

[45] Alexandrov T. , Stefanov D. , and Kamenov P. , et al.Surveillance of Foot-and-Mouth Disease (FMD) in Susceptible Wildlife and Domestic Ungulates in Southeast of Bulgaria Following a FMD Case in Wild Boar, Veterinary Microbiology. (2013) 166, no. 1-2, 84–90, 10.1016/j.vetmic.2013.05.016. 23830685

[46] Frölich K. , Hamblin C. , Parida S. , Tuppurainen E. , and Schettler E. , Serological Survey for Potential Disease Agents of Free-Ranging Cervids in Six Selected National Parks From Germany, Journal of Wildlife Diseases. (2006) 42, no. 4, 836–843, 10.7589/0090-3558-42.4.836. 17255452

[47] Marcoppido G. , Parreño V. , and Vilá B. , Antibodies to Pathogenic Livestock Viruses in a Wild Vicuña (*Vicugna vicugna*) Population in the Argentinean Andean Altiplano, Journal of Wildlife Diseases. (2010) 46, no. 2, 608–614, 10.7589/0090-3558-46.2.608. 20688660

[48] Flueck W. T. and Smith-Flueck J. A. M. , Diseases of Red Deer Introduced to Patagonia and Implications for Native Ungulates, Animal Production Science. (2012) 52, no. 8, 766–773, 10.1071/AN11342.

[49] New Zealand Government, Ministry for Primary Industries , Foot and Mouth Disease Management Strategy, 2023, Wellington, New Zealand, Accessed September 24, 2026 https://www.mpi.govt.nz/dmsdocument/58324-FMD-Disease-Managment-Strategy-2023.

[50] Australian Government, Animal Health Australia, Response Strategy , Foot-and-Mouth Disease (Version 5.0), 2022, 5th edition, Australian Veterinary Emergency Plan (AUSVETPLAN), Canberra, ACT, Accessed September 24 2026 https://animalhealthaustralia.com.au.

[51] World Organisation for Animal Health , South-East Asia and China Foot and Mouth Disease Campaign Roadmap 2021-2025, 2020, Accessed September 24, 2026 https://rr-asia.woah.org/en/seacfmd-roadmaps/.

[52] Government of the United States of America, United States Department of Agriculture , Foot-and-Mouth Disease Response Plan The Red Book, 2020, USA, Accessed September 24 2026 https://www.aphis.usda.gov/animal-emergencies/fadprep.

[53] European Union , Regulation (EU) 2016/429 of the European Parliament and of the Council of 9, on Transmissible Animal Diseases and Amending and Repealing Certain Acts in the Area of Animal Health (Animal Health Law), Official Journal of the European Union, 2016, March 2016.

[54] Ciliberti A. , Gavier-Widén D. , Yon L. , Hutchings M. R. , and Artois M. , Prioritisation of Wildlife Pathogens to Be Targeted in European Surveillance Programmes: Expert-Based Risk Analysis Focus on Ruminants, Preventive Veterinary Medicine. (2015) 118, no. 4, 271–284, 10.1016/j.prevetmed.2014.11.021. 25496774

[55] Government of the United Kingdom Department for Environment Food and Rural Affairs , Contingency Plan for Exotic Notifiable Diseases of Animals in England, 2024, United Kingdom, https://www.gov.uk/official-documents.

[56] World Organisation for Animal Health, Food and Agriculture Organization , The Global Foot And Mouth Disease Control Strategy, 2012, Accessed 2 Sep, 2026, https://www.fao.org/eufmd/global-situation/global-fmd-control-strategy/en/.

[57] EFSA Panel on Animal Health and Welfare , Scientific Opinion on Foot-and-Mouth Disease in Thrace, EFSA Journal. (2012) 10, no. 4, 10.2903/j.efsa.2012.2635. PMC13136857 42088891

[58] Brown V. R. and Bevins S. N. , Potential Role of Wildlife in the USA in the Event of a Foot-and-Mouth Disease Virus Incursion, Veterinary Record. (2019) 184, no. 24, 10.1136/vr.104895, 741. 31023873

[59] Mouchantat S. , Haas B. , and Böhle W. , et al.Proof of Principle: Non-Invasive Sampling for Early Detection of Foot-and-Mouth Disease Virus Infection in Wild Boar Using a Rope-in-a-Bait Sampling Technique, Veterinary Microbiology. (2014) 172, no. 1-2, 329–333, 10.1016/j.vetmic.2014.05.021. 24930983

[60] Giacinti J. A. , Parmley E. J. , Reist M. , Bayley D. , Pearl D. L. , and Jardine C. M. , Canadian Wildlife Health Surveillance—Patterns, Challenges and Opportunities Identified by a Scoping Review, Facets. (2022) 7, 25–44, 10.1139/facets-2021-0027.

[61] Dyason E. , Summary of Foot-and-Mouth Disease Outbreaks Reported in and Around the Kruger National Park, South Africa, Between 1970 and 2009, Journal of the South African Veterinary Association. (2010) 81, no. 4, 201–206, 10.4102/jsava.v81i4.148. 21526733

[62] Maree F. , Kasanga C. , and Scott K. , et al.Challenges and Prospects for the Control of Foot-and-Mouth Disease: an African Perspective, Veterinary Medicine: Research and Reports. (2014) 5, 119–138, 10.2147/vmrr.s62607. PMC7337166 32670853

[63] Highfield L. D. , Ward M. P. , Laffan S. W. , Norby B. , and Wagner G. G. , The Impact of Potential Mitigation Strategies on the Predicted Spread of Foot and Mouth Disease in White-Tailed Deer in South Texas, Preventive Veterinary Medicine. (2010) 94, no. 3-4, 282–288, 10.1016/j.prevetmed.2010.01.015. 20181400

[64] Griffin J. M. and O’Reilly P. J. , Epidemiology and Control of an Outbreak of Foot-and-Mouth Disease in the Republic of Ireland in 2001, Veterinary Record. (2003) 152, no. 23, 705–712, 10.1136/vr.152.23.705. 12825703

[65] European Commission DG Health and Consumers , Foot and Mouth Disease (FMD) Bulgaria 2011: Outbreak Chronology of Main Events and List of Decisions Adopted by the European Commission, 2011, Accessed May 14 2025 https://food.ec.europa.eu/document/download/a05a0366-02a7-4aeb-a120-24fb59e846d4_en?filename=ad_control-measures_fmd_chronology-bg-2011_20111109.pdf.

[66] Ivanov Y. , Alexandrov T. , Khomenko S. , and Sumption K. , Specificity of FMD Surveillance in Wild Boars, 2016, in Open Session of the European Commission for the Control of Foot-and-Mouth Disease (EuFMD), Cascais, Portugal, October 26–28, Accessed September 24 2026 https://openknowledge.fao.org/server/api/core/bitstreams/e6c4fece-8283-4582-b95a-19fde5389778/content.

[67] Chudý M. , Foot and Mouth Disease in Slovakia – State of Play, 2025, Accessed September 24 2026 https://food.ec.europa.eu/document/download/f1e84463-0a63-43cc-9215-a1be794d417c_en?filename=reg-com_ahw_20250324_pres-01.pdf.

[68] European Commission , Foot and Mouth Disease (FMD) in Germany, 2025, Accessed September 24, 2026 https://food.ec.europa.eu/animals/animal-diseases/diseases-and-control-measures/foot-and-mouth-disease_en#FMD-DE.

[69] Government of the United Kingdom, Department for Environment Food and Rural Affairs , Foot and Mouth Disease Control Strategy for Great Britain, United Kingdom, 2011, Accessed September 24, 2026 https://www.gov.uk/government/publications/foot-and-mouth-disease-control-strategy-for-great-britain.

[70] Government of the United Kingdom, Department of Agriculture , Foot and Mouth Disease Control Strategy for Northern Ireland, 2016, United Kingdom, Accessed September 24, 2026 https://www.daera-ni.gov.uk/sites/default/files/publications/dard/foot-and-mouth-disease-control-strategy-may-2016.PDF.

[71] Government of Brazil Livestock and Food Supply Secretariat for Agricultural Defense Animal Health Department , Contingency Plan For FMD - Tactical and Operation Levels, 2020, Brasil, Accessed September 24, 2026 https://www.gov.br/agricultura/pt-br/assuntos/sanidade-animal-e-vegetal/saude-animal/programas-de-saude-animal/febre-aftosa/manuais-e-relatorios/plano_contingencia_fa_ing-defeso.pdf.

[72] Fountain J. , Woodgate R. , Rast L. , and Hernández-Jover M. , Assessing Biosecurity Risks for the Introduction and Spread of Diseases Among Commercial Sheep Properties in New South Wales, Australia, Using Foot-and-Mouth Disease as a Case Study, Frontiers in Veterinary Science. (2018) 5, 10.3389/fvets.2018.00080, 80. PMC5932351 29755989

[73] Jori F. and Etter E. , Transmission of Foot and Mouth Disease at the Wildlife/Livestock Interface of the Kruger National Park, South Africa: Can the Risk Be Mitigated?, Preventive Veterinary Medicine. (2016) 126, 19–29, 10.1016/j.prevetmed.2016.01.016. 26848115

[74] Kaszta Z. , Cushman S. , Sillero-Zubiri C. , Wolff E. , and Marino J. , Where Buffalo and Cattle Meet: Modelling Interspecific Contact Risk Using Cumulative Resistant Kernels, Ecography. (2018) 41, no. 10, 1616–1626, 10.1111/ecog.03039.

[75] Niedbalski W. , Fitzner A. , and Kesy A. , Foot and Mouth Disease and Peste Des Petits Ruminants – The Role of Wildlife in the Epidemiology and Control of Diseases, Medycyna Weterynaryjna-Veterinary Medicine-Science and Practice. (2023) 79, no. 1, 111–116, 10.21521/mw.6737.

[76] Marufu M. C. , Karama M. , and Mwale-Manjoro M. , et al.SAGE Advisory on the Outbreak of Foot-and-Mouth Disease in KwaZulu-Natalin June-July 2021: Mitigating a Potential Emergency, 2021, South Africa, Accessed September 24, 2026 https://www.assaf.org.za/science-advisory-group-on-emergencies-sage/.

[77] Füssel A.-E. , Alexandrov T. , and Depner K. , Foot-and-Mouth Disease EUVET Mission to Brandenburg and Berlin Germany, 2025, Accessed September 24, 2026 https://food.ec.europa.eu/document/download/7f739215-d5a8-4448-8520-ffc05d4d75a4_en?filename=reg-com_ahw_20250122_pres-12.pdf.

[78] Cowled B. and Garner G. , A Review of Geospatial and Ecological Factors Affecting Disease Spread in Wild Pigs: Considerations for Models of Foot-and-Mouth Disease Spread, Preventive Veterinary Medicine. (2008) 87, no. 3-4, 197–212, 10.1016/j.prevetmed.2008.03.012. 18508144

[79] Du P. B. , Moswa-Kato P. , Strydom S. , Dippenaar J. , and Mabaso C. , Procedural Manual for Foot and Mouth Disease Control in South Africa: 2011, Disease Control and Epidemiology Sub-Directorates, Department Agriculture Forestry and Fisheries Mpumalanga Veterinary Services Limpopo Department of Agriculture, 2011.

[80] Republic of Botswana Office of the Auditor General Republic of Botswana , Control Of Foot and Mouth Disease by Department of Veterinary Services, 2018, Performance Audit Report No. 3. Accessed September 24, 2026 https://www.gov.bw/sites/default/files/2020-03/CONTROL%20OF%20FOOT%20AND%20MOUTH.pdf.

[81] Ferguson K. , Cleaveland S. , and Haydon D. , et al.Evaluating the Potential for the Environmentally Sustainable Control of Foot and Mouth Disease in Sub-Saharan Africa, EcoHealth. (2013) 10, no. 3, 314–322, 10.1007/s10393-013-0850-6. PMC3857521 23797715

[82] DuToit R. , Osofsky S.A. , Cleaveland S. , Karesh W.B. , Kock M.D. , Nyhus P.J. , Starr L. , and Yang A. , Foot and Mouth Disease Management and Land-use Implications in the Zimbabwean Lowveld: The Rationale for Creating a Biosphere Reserve, Conservation and Development Interventions at the Wildlife/Livestock Interface: Implications for Wildlife, Livestock and Human Health, 2005, IUCN Publications Services Unit, Gland, Switzerland and Cambridge, United Kingdom, 109–111.

[83] Guerrini L. , Pfukenyi D. , and Etter E. , et al.Spatial and Seasonal Patterns of FMD Primary Outbreaks in Cattle in Zimbabwe Between 1931 and 2016, Veterinary Research. (2019) 50, no. 1, 10.1186/s13567-019-0690-7, 73. PMC6760110 31551078

[84] Miguel E. , Grosbois V. , and Caron A. , et al.Contacts and Foot and Mouth Disease Transmission From Wild to Domestic Bovines in Africa, Ecosphere. (2013) 4, no. 4, 1–32, 10.1890/ES12-00239.1.

[85] Khanyari M. , Robinson S. , and Morgan E. , et al.Building an Ecologically Founded Disease Risk Prioritization Framework for Migratory Wildlife Species Based on Contact With Livestock, Journal of Applied Ecology. (2021) 58, no. 9, 1838–1853, 10.1111/1365-2664.13937.

[86] Bastos A. , Boshoff C. , Keet D. , Bengis R. , and Thomson G. , Natural Transmission of Foot-and-Mouth Disease Virus Between African Buffalo (*Syncerus caffer*) and Impala (*Aepyceros melampus*) in the Kruger National Park, South Africa, Epidemiology and Infection. (2000) 124, no. 3, 591–598, 10.1017/S0950268899004008. PMC2810945 10982083

[87] Omondi G. , Gakuya F. , and Arzt J. , et al.The Role of African Buffalo in the Epidemiology of Foot-and-Mouth Disease in Sympatric Cattle and Buffalo Populations in Kenya, Transboundary and Emerging Diseases. (2020) 67, 2206–2221, 10.1111/tbed.13573. 32303117

[88] Wekesa S. , Sangula A. , and Belsham G. , et al.Characterisation of Recent Foot-and-Mouth Disease Viruses From African Buffalo (*Syncerus caffer*) and Cattle in Kenya is Consistent With Independent Virus Populations, BMC Veterinary Research. (2015) 11, no. 1, 10.1186/s12917-015-0333-9, 17. PMC4334418 25644407

[89] Vosloo W. , Boshoff K. , Dwarka R. , and Bastos A. , The Possible Role that Buffalo Played in the Recent Outbreaks of Foot-and-Mouth Disease in South Africa, Annals of the New York Academy of Sciences. (2002) 969, no. 1, 187–190, 10.1111/j.1749-6632.2002.tb04376.x. 12381589

[90] Government of the United States of America, United States Department of Agriculture Animal and Plant Health Inspection Service , Report on the Review of Namibia’s Foot-and-Mouth Disease Status,, 2022, USA, Accessed September 24 2026 https://www.aphis.usda.gov/sites/default/files/namibia-status-review.pdf.

[91] Sirdar M. , Fosgate G. , and Blignaut B. , et al.A Comparison of Risk Factor Investigation and Experts’ Opinion Elicitation Analysis for Identifying Foot-and-Mouth Disease (FMD) High-Risk Areas Within the FMD Protection Zone of South Africa (2007–2016), Preventive Veterinary Medicine. (2024) 226, 10.1016/j.prevetmed.2024.106192, 106192. 38564991

[92] Dion E. , VanSchalkwyk L. , and Lambin E. F. , The Landscape Epidemiology of Foot-and-Mouth Disease in South Africa: A Spatially Explicit Multi-Agent Simulation, Ecological Modelling. (2011) 222, no. 13, 2059–2072, 10.1016/j.ecolmodel.2011.03.026.

[93] Jori F. , Vosloo W. , and Plessis B. D. , et al.A Qualitative Risk Assessment of Factors Contributing to Foot and Mouth Disease Outbreaks in Cattle Along the Western Boundary of the Kruger National Park, Revue Scientifique Et Technique De l’oie. (2009) 28, no. 3, 917–931, 10.20506/rst.28.3.1932. 20462150

[94] Mogotsi K. , Kgosikoma O. E. , and Lubinda K. F. , Wildlife-Livestock Interface, Veterinary Cordon Fence Damage, Lack of Protection Zones, Livestock Theft and Owner Apathy: Complex Socio-Ecological Dynamics in Foot and Mouth Disease Control in Southern Africa, Pastoralism Research Policy and Practice. (2016) 6, no. 1, 10.1186/s13570-016-0068-7.

[95] Sutmoller P. , Thomson G. R. , Hargreaves S. K. , Foggin C. M. , and Anderson E. C. , The Foot-and-Mouth Disease Risk Posed by African Buffalo Within Wildlife Conservancies to the Cattle Industry of Zimbabwe, Preventive Veterinary Medicine. (2000) 44, no. 1-2, 43–60, 10.1016/S0167-5877(99)00109-9. 10727743

[96] Caron A. , Miguel E. , and Gomo C. , et al.Relationship Between Burden of Infection in Ungulate Populations and Wildlife/Livestock Interfaces, Epidemiology and Infection. (2013) 141, no. 7, 1522–1535, 10.1017/S0950268813000204. PMC9151594 23442901

[97] Vosloo W. and Thomson G. , Sobrino F. and Domino E. , Natural Habitats in Which Foot-and-Mouth Disease Viruses are Maintained, Foot-and-Mouth Disease Virus: Current Research and Emerging Trends, 2017, Caister Academic Press, Norfolk, United Kingdom.

[98] Vosloo W. , Bastos A. , Sahle M. , Sangare O. , and Dwarka R. , Osofsky S.A. , Cleaveland S. , Karesh W.B. , Kock M.D. , Nyhus P.J. , Starr L. , and Yang A. , Virus Topotypes and the Role of Wildlife in Foot and Mouth Disease in Africa, Conservation and Development Interventions at the Wildlife/Livestock Interface: Implications for Wildlife, Livestock and Human Health, 2005, IUCN Publications Services Unit, Gland, Switzerland and Cambridge, United Kingdom.

[99] Hampson K. and Haydon D. , Persistent Pathogens and Wildlife Reservoirs, Science. (2021) 374, 35–36, 10.1126/science.abl8885. PMC7612424 34591640

[100] Kasanga C. J. , Dwarka R. , and Thobokwe G. , et al.Molecular Biological Characteristics of Foot-and-Mouth Disease Virus in the African Buffalo in Southern Africa, Onderstepoort Journal of Veterinary Research. (2014) 81, no. 2, 10.4102/ojvr.v81i2.728. 28235272

[101] Udahemuka J. , Aboge G. , and Obiero G. , et al.Investigation of Foot and Mouth Disease Virus and Other Animal Pathogens in Cattle, Buffaloes and Goats at the Interface With Akagera National Park 2017-2020, BMC Veterinary Research. (2022) 18, no. 1, 10.1186/s12917-022-03430-1. PMC9479285 36114497

[102] Stephen C. , The Pan-Canadian Approach to Wildlife Health, Canadian Veterinary Journal. (2019) 60, 145–146. PMC6340262 30705448

[103] Shury T. K. , Nishi J. S. , Elkin B. T. , and Wobeser G. A. , Tuberculosis and Brucellosis in Wood Bison (*Bison bison* Athabascae) in Northern Canada: A Renewed Need to Develop Options for Future Management, Journal of Wildlife Diseases. (2015) 51, no. 3, 543–554, 10.7589/2014-06-167. 25973624

[104] Aschim R. A. and Brook R. K. , Evaluating Cost-Effective Methods for Rapid and Repeatable National Scale Detection and Mapping of Invasive Species Spread, Scientific Reports. (2019) 9, no. 1, 10.1038/s41598-019-43729-y, 7254. PMC6510748 31076638

