# Supplementary material for: A Scoping Review of Foot‐and‐Mouth Disease in Wildlife: Global Evidence to Inform Preparedness in Nonendemic Regions
Source: Transbound Emerg Dis. 2026 Nov 1;2026:9482115. doi: 10.1155/tbed/9482115 (PMC13723760; doi:10.1155/tbed/9482115)
Supplement: Supplementary file 1 [file TBED-2026-9482115-s001.docx]

# Supplementary Materials

# Protocol for a scoping systematic literature review and narrative synthesis of Foot and Mouth Disease in wild animals

**Rationale**

In Canada, the Canadian Food Inspection Agency (CFIA) has prioritized many diseases of concern in order to protect domestic livestock. One such disease is Foot and Mouth Disease (FMD), a federally reportable disease in Canada.

As Canada prepares for a FMD incursion, there is a need to understand the epidemiological and ecological characteristics of FMD and prevention and control options from all perspectives to best protect the animal production sector, food sovereignty, and the health and biodiversity of our wild animals.

A literature review of FMD in wild animal populations will generate a detailed map of the knowledge base around epidemiological, ecological, and control factors, and chart options for preparedness in Canada.

**Objectives**

The broad research question guiding the review was: *‘What is the susceptibility of wildlife species to Foot and Mouth Disease and the strategies used or proposed in a response to FMD in a wildlife population?’*

The overall objective of this literature review was to identify and summarize the empirical evidence across international jurisdictions of the susceptibility of wildlife to FMD and the strategies used or proposed in a response to Foot and Mouth Disease in a wildlife population. These objectives were achieved by answering the following questions: 1) What international jurisdictions have reported on interventions related to FMD in wildlife, and what primary research studies or surveys have been conducted on FMD in wild animal hosts? And, 2) What are the surveillance, monitoring, and disease control actions described in the literature?

**Methods**

Eligibility Criteria

Studies were eligible if they:

- Are original scientific reports or reviews of findings (*i.e.* primary publications) that address the role or impact of FMD in target animal populations, or the control of FMD in target animal populations OR
- Publications (reports, websites, etc..) from international jurisdictions that describe policies, plans, recorded impact, or interventions regarding the control of FMD in target animal populations;
- Have been published in English or French;
- Target animal populations are defined as any domesticated or non-domesticated species that is free-ranging and does not depend on humans for food or reproduction (“wildlife”);
- Were published between January 1, 2000 and May 14, 2025.

Information sources

The following electronic databases were searched

- PubMed via NCBI
- Web of Science
- Agricola via Proquest
- CAB direct
- Scopus

The grey literature was searched to capture relevant non-peer-reviewed records, especially those from international jurisdictions, such as FMD contingency plans. EG searched the websites of international jurisdictions and intergovernmental organizations (e.g. Food and Agriculture Organization (FAO), the European Commission for the control of Foot and Mouth Disease (EuFMD), and the Pan American Center for Foot-and-Mouth Disease (PANAFTOSA)) and Google between 8 April – 14 May, 2025 using tailored keyword strings in English like those used in the academic database searches. Results were documented in an excel workbook.

Search Strategy

The search was limited to the year 2000 or later. Search terms and strategies were tailored to the requirements and structure of each database, and consisted of combinations of pathogen and host (Table S1).

Search term categories:

Disease:

- Foot and Mouth Disease
- FMD
- Hoof and mouth disease
- Foot and Mouth Disease virus
- Foot-and-Mouth Disease virus
- FMDV

Hosts:

- Wild*
- Free-rang*
- Feral

Table S1. Electronic database search results for a scoping literature review exploring the role of wildlife in the epidemiology of Foot and Mouth disease.

| Database | Search query | Search date | Number of records | Search Notes |
| --- | --- | --- | --- | --- |
| PubMed | ((FMD) OR (FMDV) OR (Foot and mouth disease) OR (Hoof and mouth disease) OR ( Foot-and-Mouth disease)OR ( Foot-and-Mouth Disease virus) OR(Foot and Mouth Disease virus)) AND (Wild* OR Free-ranging OR Feral) | 28 March 2025 | 660 |  |
| Web of Science | Foot and Mouth Disease (All Fields) or FMD (All Fields) or Hoof and mouth disease (All Fields) or Foot and Mouth Disease virus (All Fields) or foot-and-mouth disease virus (All Fields) or FMDV (All Fields) AND ((ALL=(wild*)) OR ALL=(free-rang*)) OR ALL=(feral) | 4 April 2025 | 989 | Searched FMD keywords (query 1) then Wildlife keywords (query 2) then combined with AND for new result; then limited by 2000 or newer. |
| Scopus | ( ( TITLE-ABS-KEY ( wild* ) OR TITLE-ABS-KEY ( free-rang* ) OR TITLE-ABS-KEY ( feral ) ) ) AND ( ( TITLE-ABS-KEY ( foot AND mouth AND disease ) OR TITLE-ABS-KEY ( fmd ) OR TITLE-ABS-KEY ( hoof AND mouth AND disease ) OR TITLE-ABS-KEY ( foot AND mouth AND disease AND virus ) OR TITLE-ABS-KEY ( foot-and-mouth AND disease AND virus ) OR TITLE-ABS-KEY ( fmdv ) ) ) | 4 April 2025 | 804 | Searched title, abstract, keyword; then limited by 2000 or newer; then limited to English/French |
| Biosis | (((((TS=(Foot and Mouth Disease)) OR TS=(FMD)) OR TS=(Hoof and mouth disease)) OR TS=(Foot and Mouth Disease virus)) OR TS=(foot-and-mouth disease virus)) OR TS=(FMDV) AND ((TS=(wild*)) OR TS=(free-rang*)) OR TS=(feral) | 4 April 2025 | 518 | Searched FMD keywords (query 1) then Wildlife keywords (query 2) then combined with AND for new result; then limited by 2000 or newer. |

Table S2. Grey literature search results for a scoping literature review exploring the role of wildlife in the epidemiology of Foot and Mouth disease.

| Website or jurisdiction | Search date in 2025 | No. of records included in full-text screening |
| --- | --- | --- |
| FAO | 8-Apr | 9 |
| EUFMD quarterly reports | 8 Apr | 66 |
| Bulgaria ministry of Agriculture | 29-Apr | 2 |
| UK DEFRA | 30-Apr | 5 |
| Germany Federal Office for agriculture and food (BLE) | 30-Apr | 0 |
| Turkiye Directorate of Food and Control | 30-Apr | 0 |
| European Commission | 30-Apr | 19 |
| South Africa Department of Agriculture, Land, and rural development | 1-May | 0 |
| google.com | 2-May | 16 |
| PANAFTOSA | 2-May | 2 |
| United States Department of Agriculture | 14-May | 2 |
| Canadian Food Inspection Agency | 14 May | 0 |

Data Management

Search results were uploaded into Covidence reference management software and duplicates removed.

Study Selection

Title and abstract screening and full-text screening and data characterization was completed by EG, JR and EC.

Title and abstract screening: Inclusion/Exclusion criteria

1. Does this publication report on FMD in wild animals, defined as any domesticated or non-domesticated species that is free-ranging and does not depend on humans for food or reproduction?

- Yes
- No (do not continue)

2. Does this publication report:
   a. clinical or epidemiological characteristics and impact AND/OR
   b. control measures or interventions?

- Yes
- No (do not continue)

3. Is this primary research, a review, opinion, or jurisdictional publication since 2000?

- Yes
- No (do not continue)

Full text screening and data characterization:

## 1. **Full text screening**

Table S3. Inclusion and exclusion criteria used for full text screening for a systematic literature review exploring the role of wildlife in the epidemiology of Foot and Mouth disease.

|  | Include | Exclude |
| --- | --- | --- |
| Population | Wild animals: Any domesticated or non-domesticated species that is free-ranging and does not depend on humans for food or reproduction | Domestic animals  Zoo animals or captive wild animals |
| Disease/Pathogen | Foot and mouth disease virus |  |
| Outcome | Clinical or epidemiological characteristics and impact  Control measures or interventions |  |
| Study Characteristics^[1]^ | Published within or after 2000  Observational study  Experimental study  Modelling study  Report  Commentary/opinion  Narrative review | Formal or systematic review |

# 2. Data extraction

Table S4. Categories and informational fields used for data extraction of the included publications for a systematic literature review exploring the role of wildlife in the epidemiology of Foot and Mouth disease. Data extraction was organized around categories related to the study objectives.

| Category | Extraction fields |
| --- | --- |
| General Information | Title, publication year, corresponding author, country/region study was conducted in, disclosed conflict of interest, publication type, aim of publication |
| Susceptibility | Species/animal type, how susceptibility was measured, if susceptibility was found, clinical signs, summary |
| Sampling | Sampling strategy, size of target population, sample size, specimens collected, collection method, diagnostic tests |
| Surveillance | Implemented or planned, surveillance type, objectives, conclusions/findings, advantages or challenges described |
| Control Measures | Type of control method, summary of methods, advantages or challenges described |
| Other questions | Ecological role, mechanical vectors, role of climate change, cost/benefit of wildlife interventions, impact on wildlife, inclusion of wildlife groups in contingency planning |

**PRISMA flow diagram for a literature review of FMD in wildlife**

Studies from databases/registers **(n = 3092)**

Web of Science (n = 989)

Scopus (n = 804)

PubMed (n = 660)

BIOSIS (n = 518)

Grey literature (n = 121)

References removed **(n = 1636)**

Duplicates identified manually (n = 16)

Duplicates identified by Covidence (n = 1620)

**Identification**

Studies included in review **(n = 205)**

Studies not retrieved **(n = 0)**

Studies assessed for eligibility **(n = 429)**

Studies sought for retrieval **(n = 429)**

Studies screened **(n = 1456)**

Studies excluded **(n = 1027)**

Studies excluded **(n = 224)**

Textbook (n = 6)

no full text (n = 9)

Review article (n = 17)

Wrong pathogen (n = 4)

captive wild population (n = 8)

outside publication date (n = 1)

Domestic animal population (n = 79)

translation unavailable or poor (n = 4)

No discussion of outcome criteria (n = 96)

**Screening**

**Included**

Figure S1. Prisma flow diagram for the literature review of FMD in wildlife.

1. Commentaries, opinion pieces, and narrative reviews were included to capture recommendations around how to address FMD in wildlife. Systematic reviews were excluded to prevent duplication of results.
